# Sunlight-driven and gram-scale vanillin production *via* Mn-defected γ-MnO_2_ catalyst in aqueous environment†

**DOI:** 10.1039/d3sc05654f

**Published:** 2024-02-19

**Authors:** Qingping Ke, Yurong Zhang, Chao Wan, Jun Tang, Shenglai Li, Xu Guo, Minsu Han, Takashi Hamada, Sameh M. Osman, Yunqing Kang, Yusuke Yamauchi

**Affiliations:** a School of Chemistry and Chemical Engineering, Anhui University of Technology Ma'anshan 243002 China wanchao@zju.edu.cn; b College of Chemical and Biological Engineering, Zhejiang University Hangzhou 310058 China; c Department of Materials Science and Chemical Engineering, Stony Brook University New York 11794 USA; d Australian Institute for Bioengineering and Nanotechnology (AIBN), The University of Queensland Brisbane Queensland 4072 Australia minsu.han@uq.edu.au; e Department of Materials Process Engineering, Graduate School of Engineering, Nagoya University Nagoya 464-8603 Japan; f Chemistry Department, College of Science, King Saud University P.O. Box 2455 Riyadh 11451 Saudi Arabia; g Research Center for Materials Nanoarchitectonics (MANA), National Institute for Materials Science (NIMS) 1-1 Namiki Tsukuba Ibaraki 305-0044 Japan yqkang@toki.waseda.jp; h Department of Chemical and Biomolecular Engineering, Yonsei University Seoul 03722 South Korea

## Abstract

The production of vanillin from biomass offers a sustainable route for synthesizing daily-use chemicals. However, achieving sunlight-driven vanillin synthesis through H_2_O activation in an aqueous environment poses challenges due to the high barrier of H_2_O dissociation. In this study, we have successfully developed an efficient approach for gram-scale vanillin synthesis in an aqueous reaction, employing Mn-defected γ-MnO_2_ as a photocatalyst at room temperature. Density functional theory calculations reveal that the presence of defective Mn species (Mn^3+^) significantly enhances the adsorption of vanillyl alcohol and H_2_O onto the surface of the γ-MnO_2_ catalyst. Hydroxyl radical (˙OH) species are formed through H_2_O activation with the assistance of sunlight, playing a pivotal role as oxygen-reactive species in the oxidation of vanillyl alcohol into vanillin. The Mn-defected γ-MnO_2_ catalyst exhibits exceptional performance, achieving up to 93.4% conversion of vanillyl alcohol and 95.7% selectivity of vanillin under sunlight. Notably, even in a laboratory setting during the daytime, the Mn-defected γ-MnO_2_ catalyst demonstrates significantly higher catalytic performance compared to the dark environment. This work presents a highly effective and promising strategy for low-cost and environmentally benign vanillin synthesis.

## Abstract 2

We demonstrate Mn-defected γ-MnO_2_ as photocatalyst for synthesis of vanillin in aqueous environment under room temperature. The ˙OH species on Mn-defected γ-MnO_2_ act as reactive oxygen species for oxidation of vanillyl alcohol into vanillin.

## Graphical abstract

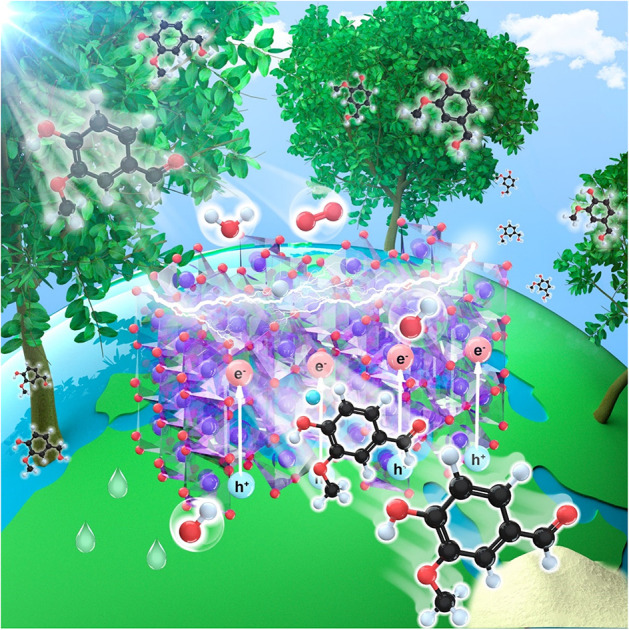

## Introduction

Vanillin, a highly popular spice worldwide, is extensively used in food additives, perfumes, and daily-use chemicals.^1^ Traditionally, it is derived from fossil fuels through the Solvay route,^2^ which involves the oxidation of vanillyl alcohol to vanillin using methanol as a solvent. In addition to the Solvay route, about 15% of vanillin is directly isolated from depolymerized lignin derivatives through upcycling,^3–5^ presenting an eco-friendly method that avoids reliance on fossil fuels. However, only approximately 2% of lignin currently undergoes conversion to value-added chemicals, with the remaining 98% being incinerated for energy in the pulp and paper industry. Consequently, a significant challenge in terms of sustainability and environmental protection lies in the valorization of lignin.^6–9^ Among potential approaches, the synthetic vanillin market, producing around 17 000 tons per year, primarily relies on fossil-based routes. Chemical conversions of lignin-derived compounds, such as vanillyl alcohol, offer a sustainable and cost-effective route to replenish the synthetic vanillin market.^10^ Unfortunately, most lignin-to-vanillin processes operate in organic solvents, requiring high temperature/O_2_ pressure,^6^ and consuming artificial energy due to recrystallization and solvent extraction steps.^11^ In response to these drawbacks, the development of sunlight-driven organic transformations through the construction of novel heterogeneous catalyst materials that are efficient and separable even in aqueous reactions holds promise for the sustainable synthesis of valuable chemicals.^12^

The water-participated route for alcohol oxidation signifies a noteworthy advancement in organic synthesis,^13^ offering a novel and sustainable approach to vanillin synthesis. However, this oxidation process encounters challenges due to the energy-demanding O–H bond activation of H_2_O.^14^ While light-driven H_2_O dissociation has emerged as an attractive method to activate the O–H bond, the limited success cases of this process mainly rely on noble metal catalysts and artificial light sources, such as a Xenon lamp.^15^ An effective strategy for manipulating the H_2_O dissociation pathway, including the energy requirement for O–H bond activation and the chemical nature of the active metal/oxygen species, involves the coordination engineering of metal oxide catalysts. The coordination engineering of bismuth (Bi) and oxygen (O) sites in the BiOBr catalyst has successfully demonstrated the enhancement of ethylbenzene oxidation through sunlight-driven reduction of the adsorption barrier for H_2_O activation.^15^ However, investigations into the sunlight-driven synthesis of vanillin in an aqueous environment using metal oxides remain relatively scarce.

MnO_2_, a transition metal oxide, has garnered significant attention as a heterogeneous catalyst for catalytic oxidation reactions, owing to its inherent advantages, including multivalence (Mn^2+^, Mn^3+^, and Mn^4+^)^16^ and a variable structure (α-, γ-, ε-, R-, and β-MnO_2_).^17^ Among these structures, γ-MnO_2_, characterized by a disordered structure comprising the intergrowth of β-MnO_2_ and R-MnO_2_, stands out as one of the most extensively studied manganese dioxides.^18,19^ Herein, we report the successful fabrication of a Mn-defected γ-MnO_2_ catalyst for the water-participated oxidation of vanillyl alcohol to vanillin in an aqueous reaction under sunlight illumination. Our approach offers several notable advantages (Table S1†): (1) the use of H_2_O as a green reaction medium and oxygen source, promoting environmental sustainability; (2) utilization of sunlight as the energy source for H_2_O activation, reducing reliance on artificial light sources; (3) conducting the reaction at room temperature, minimizing energy requirements, and enabling milder reaction conditions; (4) the possibility of achieving gram-scale reactions, allowing for large-scale production; (5) a separable, additive-free, and carbon-efficient protocol, enhancing the overall efficiency and sustainability of the process.

## Results and discussion

**Fig. 1:**
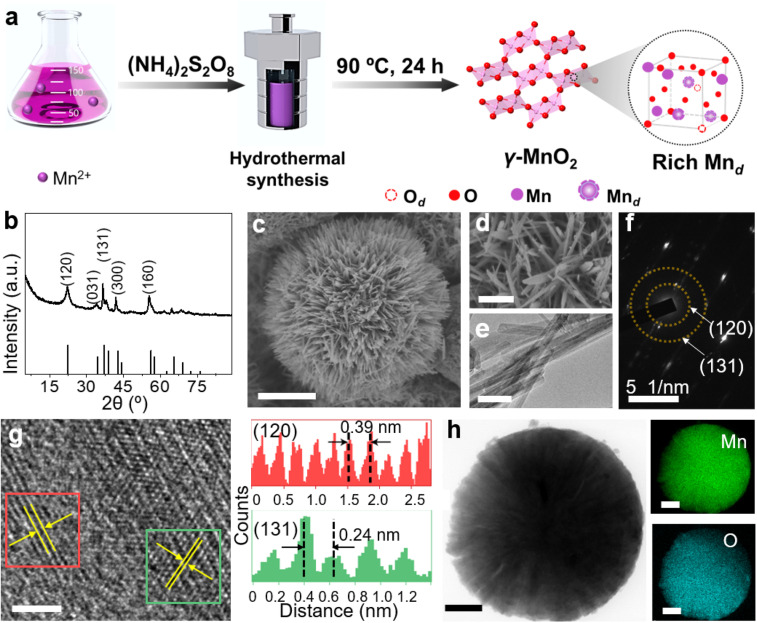
(a) Schematic synthesis process of γ-MnO_2_ catalysts with Mn defects (Mn_d_). (b) PXRD pattern, (c and d) SEM images, (e) TEM image, (f) SAED pattern, and (g) HRTEM image (left) and corresponding intensity plot (right) of γ-MnO_2_ catalyst. (h) EDX elemental maps showing the distributions of Mn and O. Scale bars: (c) 2 μm, (d) 500 nm, (e) 50 nm, (g) 2 nm, (h) 1 μm.

γ-MnO_2_ catalysts with defects in Mn species were obtained through a hydrothermal process (Fig. 1a, see ESI† for preparation details). The crystal structures of the as-prepared γ-MnO_2_ catalysts were characterized using powder X-ray diffraction (PXRD). As shown in Fig. 1b and S1,† regardless of the amount of urea, the synthesized γ-MnO_2_, γ-MnO_2_(1), and γ-MnO_2_(10) catalysts (1 and 10 referring to the feeding amount of urea) exhibit peaks at 2*θ* = 22.4°, 37.0°, 42.3°, and 56.0°, corresponding to the (120), (131), (300), and (160) planes, respectively. These planes are well indexed to the layered γ-MnO_2_ (JCPDS-14-0644).^20,21^ For comparison, MnO_2_ catalysts with different crystal structures, such as α-MnO_2_, β-MnO_2_, and ε-MnO_2_, were synthesized using different raw materials^22^ and characterized by PXRD (Fig. S2†). Scanning electron microscopy (SEM) and transmission electron microscopy (TEM) analyses reveal that the as-prepared γ-MnO_2_ particles exhibit a spherical shape with a size of 6 μm (Fig. 1c), in which nanorods with a thickness of several tens of nanometers are assembled (Fig. 1d and e). In the selected area electron diffraction (SAED) pattern (Fig. 1f) and high-resolution TEM (HRTEM) image (Fig. 1g) of γ-MnO_2_ nanorods, crystalline spaces of 0.24 and 0.39 nm corresponding to the (131)^23,24^ and (120)^25,26^ planes of γ-MnO_2_, respectively, are observed, consistent with the XRD results. Energy-dispersive X-ray spectroscopy (EDS) mapping confirms the uniform dispersion of Mn and O throughout the γ-MnO_2_ particle (Fig. 1h). Notably, no N residue is observed for γ-MnO_2_ according to X-ray photoelectron spectroscopy (XPS) characterization (Fig. S3a†). Both γ-MnO_2_(1) and γ-MnO_2_(10) particles synthesized in the presence of urea exhibit a morphology observed in SEM images (Fig. S4†), a crystal structure observed in TEM images (Fig. S5†), and N_2_ adsorption–desorption isotherms (Fig. S6†) similar to γ-MnO_2_ synthesized without urea.

**Fig. 2:**
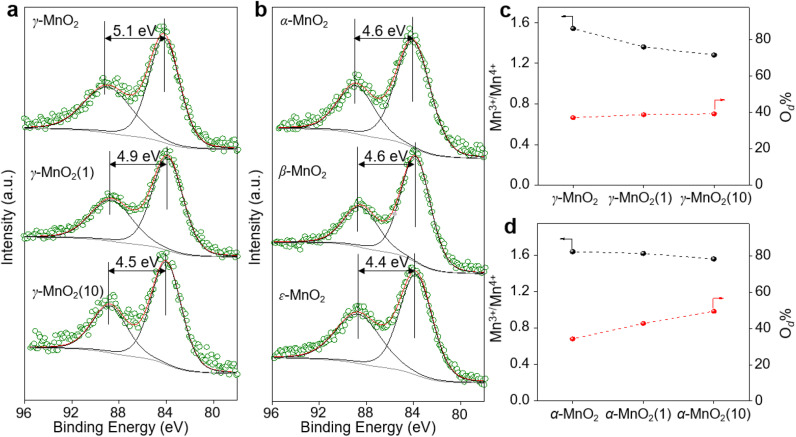
Mn 3s XPS spectra for (a) γ-MnO_2_ with different amounts of urea added in the synthesis and (b) MnO_2_ with different crystal structures. The molar ratio of Mn^3+^/Mn^4+^ and percentage of oxygen defects (O_d_) in different (c) γ-MnO_2_ and (d) α-MnO_2_.

XPS is a regular and powerful tool used to identify the surface elemental species and electronic states of materials. Fig. 2 and S7–S10† show the Mn 3s, Mn 2p, and O 1s XPS patterns of the as-prepared α-MnO_2_, β-MnO_2_, ε-MnO_2_, and γ-MnO_2_ catalysts. The Δ*E*_s_ (binding energy between two peaks of Mn 3s multiplet splitting) of the γ-MnO_2_ catalysts in the range of 4.5–5.1 eV confirm the presence of Mn^3+^ species (Fig. 2a),^27^ indicating the coexistence of both Mn^3+^ and Mn^4+^ species in these catalysts. The average oxidation states (AOS) of Mn species are calculated using the following formula.^28,29^AOS = 8.956 − 1.126Δ*E*_s_

As shown in Fig. 2a, the calculated AOS values are 3.2, 3.4, and 3.9 for γ-MnO_2_, γ-MnO_2_(1), and γ-MnO_2_(10), respectively, consistent with the Mn^3+^/Mn^4+^ ratios (Fig. S7a–c,† Mn^3+^/Mn^4+^ = 1.54, 1.36, and 1.28 for γ-MnO_2_, γ-MnO_2_(1), and γ-MnO_2_(10) particles, respectively). It's worth noting that the oxygen defects (O_d_) remain almost constant in γ-MnO_2_, γ-MnO_2_(1), and γ-MnO_2_(10) (37.2–39.3%), as shown in Fig. S7d–f.† Interestingly, MnO_2_ with different crystal structures exhibits varying degrees of Mn defects (Mn_d_) associated with Mn^3+^ species (Fig. 2b). The calculated AOS values are 3.8, 3.8, and 4.0 for α-MnO_2_, β-MnO_2_, and ε-MnO_2_, respectively, with corresponding ratios of Mn^3+^/Mn^4+^ being 1.51, 1.50, and 1.47 (Fig. S8†). The highest ratio of Mn^3+^/Mn^4+^ (1.54) in γ-MnO_2_ among the MnO_2_ is ascribed to its most abundant Mn_d_ caused by the coordination unsaturation between the lattice oxygen and lattice Mn. Typically, unsaturated metal species and O_d_ species, which are active in various oxidation reactions, coexist in metal oxides.^30,31^ It is well known that Mn_d_, caused by coordination unsaturation between lattice oxygen and lattice Mn, can be tuned by inducing O_d_ or adjusting non-metal dopant amounts.^32–34^ Contrary to the almost constant presence of O_d_ species (Fig. 2c), significantly higher N dopants are observed in γ-MnO_2_(1) and γ-MnO_2_(10) compared to γ-MnO_2_ (Fig. S3†).^35^ The N anionic (N^3−^) dopants possess excess negative charges compared to the O anionic (O^2−^),^36^ leading to a higher AOS of Mn species due to the charge compensation for higher N dopants. A similar higher AOS of Mn species in MnO_2_ was previously observed with boron doping.^37^ For comparison, we synthesized α-MnO_2_ catalysts with different O_d_ sites. As shown in Fig. 2d and S9,† surface O_d_ sites in α-MnO_2_ catalysts significantly increase after the addition of urea during the hydrothermal process, while the Mn^3+^ species remain almost constant (Fig. S10†). The nearly unchanged ratio of Mn^3+^/Mn^4+^, despite the differing amounts of O_d_ species in α-MnO_2_(1) and α-MnO_2_(10), may be attributed to the presence of N anionic residues. The detailed mechanisms of O_d_ species formation are beyond the scope of the current stage of study and will be pursued in our future work.

**Fig. 3:**
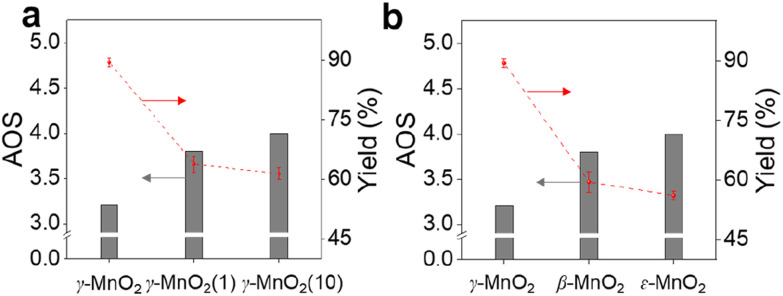
Catalytic performance of γ-MnO_2_ and comparison catalysts for oxidation of vanillyl alcohol to vanillin. (a) AOS-yield of vanillin on γ-MnO_2_, (b) AOS-yield of vanillin on MnO_2_ with various crystal structures.

To demonstrate the practicality of our approach, we conducted a gram-scale oxidation of vanillyl alcohol to vanillin in an aqueous environment as a model reaction, and the results are presented in Table 1. For the catalytic reaction, 0.77 g of vanillyl alcohol, 6.0 mL of H_2_O, and 10.0 mmol of catalyst were added to a quartz reactor and stirred at room temperature under sunlight for 10 h while exposed to air. No product is obtained in the absence of a catalyst (Table 1, entry 1). The as-prepared MnO_2_ catalysts, including α-, β-, ε-, and γ-MnO_2_ (Table 1, entries 2–9), are found to be active for the oxidation of vanillyl alcohol to vanillin. MnO_2_ catalysts with more abundant O_d_ (Fig. 2d), such as α-MnO_2_(1) and α-MnO_2_(10), exhibit lower catalytic performance compared to α-MnO_2_ with fewer O_d_, indicating that O_d_ species are not a key factor in promoting the catalytic activity of MnO_2_ catalysts for the oxidation under sunlight. The γ-MnO_2_ catalyst exhibits the highest catalytic activity and vanillin selectivity, converting vanillyl alcohol to vanillin with a yield of nearly 90% (Table 1, entry 2), outperforming the γ-MnO_2_(1) and γ-MnO_2_(10) catalysts (Table 1, entries 3,4). Since γ-MnO_2_, γ-MnO_2_(1), and γ-MnO_2_(10) catalysts have similar surface O_d_ species (Fig. 2c), Brunauer–Emmett–Teller (BET) surface areas (Fig. S6†), and surface morphologies (Fig. 1c, d and S4†), the superior catalytic performance of the γ-MnO_2_ catalyst among them can be attributed to the most abundant Mn_d_ species on the surface (Fig. 3a). Similarly, the best vanillin selectivity and superior catalytic performance displayed by γ-MnO_2_, compared to other MnO_2_ catalysts with diverse crystal structures (Table 1, entries 2–5 and 9–10), can be attributed to the prevalence of its Mn_d_ species (Fig. 3). Given the significant influence of factors, such as solvent and temperature, on the catalytic oxidation reaction of vanillyl alcohol,^38^ we explored various conditions using the γ-MnO_2_ catalyst to determine the optimal reaction parameters. The γ-MnO_2_ catalyst exhibits remarkably superior catalytic performance at 30 °C (Table S2†) and when using H_2_O as a solvent (Table S3†). The significant improvement in the catalytic performance of γ-MnO_2_ catalyst in H_2_O compared to other solvents (Table S3†) may be attributed to the role of H_2_O in activating O_2_/H_2_O to form key active O species, as discussed in the following section on the mechanism.

**Table 1:** Oxidation of vanillyl alcohol to vanillin over various catalysts

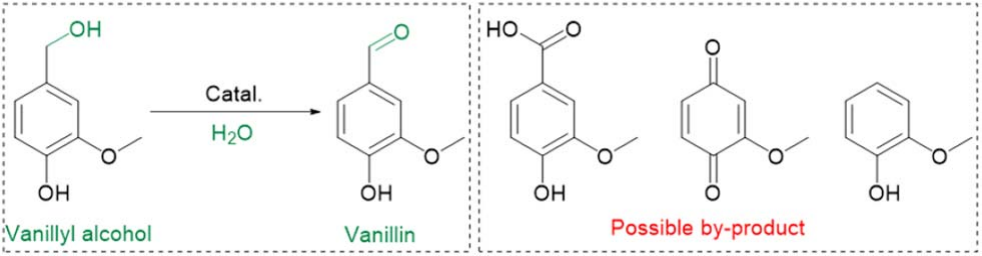				
Entry	Catalyst	Con.%	Sel.%^a^	Yield%
1	—	n.d.	—	—
2	γ-MnO_2_	93.4	95.7	89.4
3	γ-MnO_2_(1)	66.3	95.5	63.3
4	γ-MnO_2_(10)	63.3	94.9	60.1
5	α-MnO_2_	60.8	86.3	52.5
6	α-MnO_2_(1)	25.5	45.7	11.7
7	α-MnO_2_(10)	26.0	54.0	14.0
8	β-MnO_2_	63.0	94.4	59.5
9	ε-MnO_2_	61.4	91.3	56.1

a Selective generation of vanillin. Reaction conditions: vanillyl alcohol (0.77 g), Solvent (H_2_O, 6.0 mL), catalyst 10.0 mmol, open to air, sunlight, 30 °C, 10 h. (n.d. = Not detected).

**Fig. 4:**
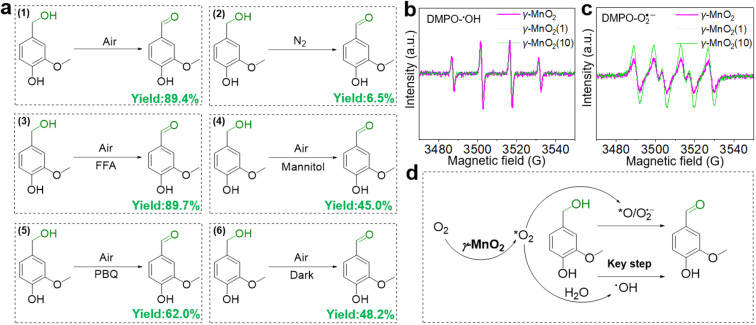
Exploring the role of oxygen species in the oxidation of vanillyl alcohol over γ-MnO_2_ catalyst. (a) Oxidation of vanillyl alcohol under different atmospheres or with various quenches. (b and c) EPR spectra of DMPO-˙OH and DMPO-O_2_˙^−^ over various γ-MnO_2_ catalysts. (d) Proposed the role of oxygen species in the oxidation of vanillyl alcohol. *O_2_ refers to the adsorbed oxygen.

To elucidate its superior catalytic performance, the catalytic mechanism of the oxidation of vanillyl alcohol over the γ-MnO_2_ catalyst was evaluated. In comparison to the impressive catalytic performance under O_2_ (Fig. 4a, eqn (1)), the oxidation of vanillyl alcohol is dramatically suppressed (∼6.5% yield of vanillin) when the reaction is performed under N_2_ (Fig. 4a, eqn (2)), confirming that oxidation over the γ-MnO_2_ catalyst mainly occurs through a dissolved oxygen species-mediated route.^39^ After the reaction under the N_2_ atmosphere, the ratio of surface Mn^3+^/Mn^4+^ species increases from 1.54 to 1.63 (Fig. S11†), indicating the involvement of Mn species in the catalytic cycle.

Considering the importance of oxygen in determining oxidative activity, we further elucidate the role of dissolved oxygen species in sunlight oxidation through scavenger control experiments. Despite the addition of furfuryl alcohol (FFA, an efficient scavenger of ^1^O_2_) to the reactants, the yield of vanillin is 89.4% (Fig. 4a, eqn (3)), indicating that FFA does not affect the activity of the γ-MnO_2_ catalyst. On the other hand, mannitol (an efficient scavenger of ˙OH) causes a significant decrease in the yield of vanillin (∼45.0%) (Fig. 4a, eqn (4)). With 1,4-Benzoquinone (PBQ, an efficient scavenger of O_2_˙^−^) added during the reaction, only a normal conversion inhibition of vanillyl alcohol is observed and ∼62.0% yield of vanillin is obtained (Fig. 4a, eqn (5)). The above analyses confirm that the oxidation of vanillyl alcohol to vanillin under sunlight occurs with the assistance of O_2_˙^−^ and ˙OH species derived from O_2_/H_2_O activation by the γ-MnO_2_ catalyst.

Electron paramagnetic resonance (EPR) was employed to confirm the presence of O_2_˙^−^ and ˙OH species during the oxidation process.^40^ Under the reaction conditions, a quadruple peak with an intensity ratio of 1 : 2 : 2 : 1 is observed for the characteristic peak of pyrroline nitrogen oxide (DMPO)-˙OH (Fig. 4b).^41^ Another quadruple peak with an intensity ratio of 1 : 1 : 1 : 1 is observed for the characteristic peak of DMPO-O_2_˙^−^ (Fig. 4c),^42^ indicating the formation of O_2_˙^−^ and ˙OH species. Notably, the peak intensity of DMPO-˙OH over the γ-MnO_2_ catalyst with superior catalytic activity is stronger than that of γ-MnO_2_(1) and γ-MnO_2_(10) catalysts with mediocre activity. In contrast, the intensities of DMPO-O_2_˙^−^ over the γ-MnO_2_ catalyst shows opposite trends. These results further support that ˙OH species are the key reactive oxygen species in the oxidation reaction of vanillyl alcohol *via* γ-MnO_2_ under sunlight (Fig. 4d), consistent with the results in Fig. 4a. Meanwhile, the stronger DMPO-˙OH signal for γ-MnO_2_ compared to the other two samples indicates enhanced ˙OH generation due to the rich-Mn_d_. Interestingly, under dark conditions, the γ-MnO_2_ catalyst achieves a ∼48.2% yield of vanillin (Fig. 4a, eqn (6)), similar to the 45.0% yield obtained when mannitol is added to the reaction mixture (Fig. 4a, eqn (4)). Additionally, the γ-MnO_2_ catalyst exhibits broad light absorption from ultraviolet to visible light, effectively covering most of the solar spectrum (Fig. S12†). This characteristic enables the abundant generation of ˙OH species under natural light exposure at 30 °C.

**Fig. 5:**
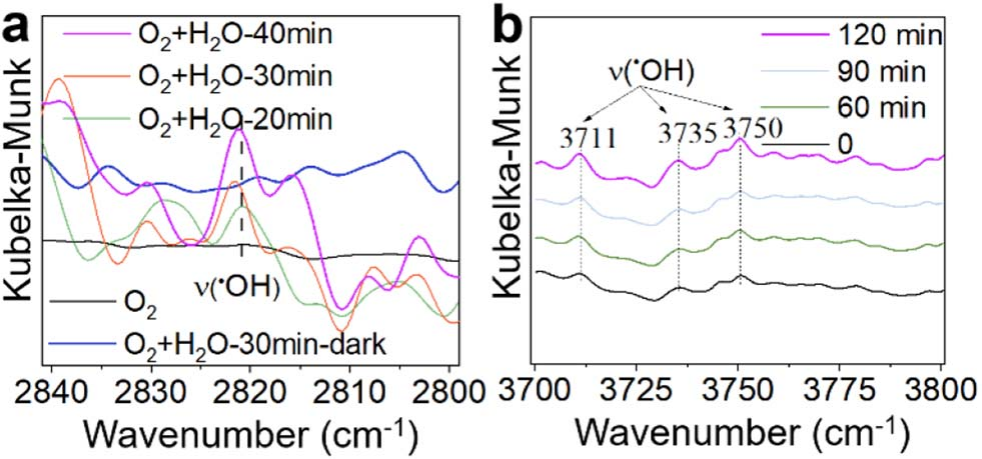
*In situ* diffuse reflectance IR spectra (DRIFTS) of activation of O_2_/H_2_O over γ-MnO_2_ catalyst. (a) Activation of O_2_/H_2_O under various conditions. (b) Activation of O_2_/H_2_O under sunlight illustration.

Based on the above analysis, O_2_, H_2_O, and sunlight emerge as key factors influencing the catalytic performance of the γ-MnO_2_ catalyst in the oxidation of vanillyl alcohol to vanillin. Previous studies have demonstrated that ˙OH species can be generated from H_2_O/O_2_ mixtures on δ-MnO_2_ or Mn/Na_2_WO_4_/SiO_2_ catalysts under sunlight conditions.^43,44^ To verify the formation of ˙OH species in H_2_O/O_2_ mixtures over the γ-MnO_2_ catalyst, *in situ* IR spectroscopy was employed, and the results are depicted in Fig. 5. The stretching vibration peaks of ˙OH species at ∼2820, 3711, 3735 and 3750 cm^−1^ intensify with prolonged sunlight illumination (Fig. 5). However, the stretching vibration of ˙OH species is not observed in the absence of H_2_O (Fig. 5a, black curve) or under dark conditions (Fig. 5a, blue curve). These results indicate that both H_2_O and sunlight play crucial roles in the formation of ˙OH species in H_2_O/O_2_ mixtures. In the oxidation reaction, oxygen is considered to be the ideal oxidant, with H_2_O and H_2_O_2_ identified as by-products. Identifying these by-products is vital for elucidating the reaction mechanism, especially as ˙OH species could be produced from H_2_O_2_ species. Further investigation into the by-product of H_2_O_2_ species was conducted using the iodometry method, and the results are presented in Fig. S13.† A peak at ∼365 nm, assigned to the formation of H_2_O_2_, is detected under sunlight illustration. However, H_2_O_2_ species are not produced under dark conditions. Similar results are obtained in the absence of a reactant or γ-MnO_2_ catalyst. These findings confirm that H_2_O_2_ species, as a by-product, are produced during the oxidation of vanillyl alcohol to vanillin over the γ-MnO_2_ catalyst with the assistance of sunlight illustration.

**Fig. 6:**
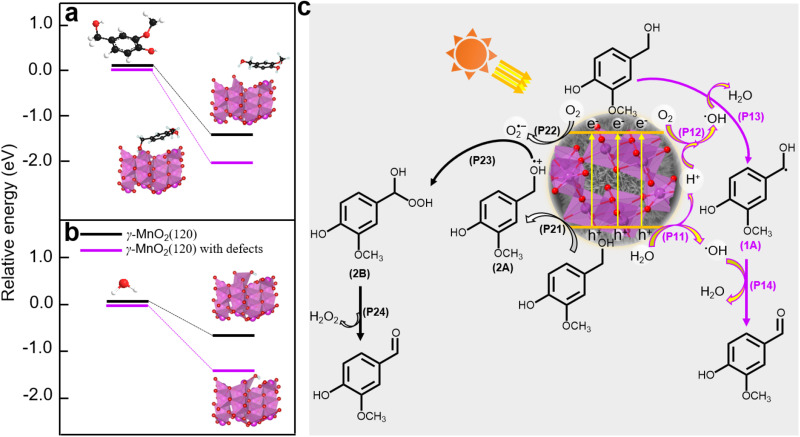
(a) DFT calculations for vanillyl alcohol adsorbed on γ-MnO_2_ catalyst with/without defects. (b) DFT calculations for H_2_O adsorbed on γ-MnO_2_ catalyst with/without defects. (c) A plausible mechanism of oxidation of vanillyl alcohol to vanillin over γ-MnO_2_ catalyst under air atmosphere.

Density functional theory (DFT) calculations were employed to elucidate the absorption sites on the γ-MnO_2_ catalyst, comprising a perfect (120) facet and defective (120) facets (Fig. S14†). The DFT calculations (Fig. 6a and b) reveal that vanillyl alcohol and H_2_O preferentially adsorb on the Mn_d_ species (Mn^3+^ species) of defective (120) facets, regardless of the presence of O_d_ species for both models. The preference of vanillyl alcohol for adsorption on the Mn_d_ species is further supported by DFT calculations in Fig. 6a and S15,† illustrating the interaction between Mn^3+^ species of MnO_2_ and vanillyl alcohol. This interaction results in an adsorption energy of −217.3 kJ mol^−1^ (Fig. S15†), significantly exceeding the adsorption energy of −131.5 kJ mol^−1^ observed for MnO_2_ featuring only O_d_ sites. Similarly, H_2_O exhibits a preference for adsorption on the Mn_d_ species rather than O_d_ sites, as depicted in Fig. 6b. Furthermore, oxygen molecules from the air can also be absorbed by the Mn_d_. Previous studies have reported the adsorption of oxygen molecules on Mn_d_ in MnO_2_ catalysts.^45^

Based on the analysis and insights gleaned from previous studies,^10,46,47^ we propose a plausible mechanism for the oxidation of vanillyl alcohol to vanillin using the γ-MnO_2_ catalyst in air (Fig. 6c). In this mechanism, O_2_, H_2_O, and vanillyl alcohol are simultaneously adsorbed onto the catalyst. Under sunlight illumination, electrons (e^−^) and holes (h^+^) are separated from the surface of the γ-MnO_2_ catalyst. During this step, adsorbed H_2_O accepts holes (h^+^) to form ˙OH species, releasing H^+^ species (P11, Fig. 6c). Simultaneously, vanillyl alcohol accepts holes (h^+^) to generate alcohol radical species (2A) (P21, Fig. 6c). Adsorbed oxygen can accept e^−^ from the γ-MnO_2_ catalyst surface to form O_2_˙^−^ species (P22, Fig. 6c), or it can combine with protons generated from P11 to form ˙OH species (P12, Fig. 6c). The formation of ˙OH species from O_2_ and H_2_O in oxide-based catalysts, including MnO_2_, has also been proposed^44,48^ Vanillyl alcohol radical (1A, Fig. 6c) is selectively obtained by combining the adsorbed vanillyl alcohol with ˙OH species (P13, Fig. 6c), which further converts to vanillin through a subsequent reaction with ˙OH species (P14, Fig. 6c). The positive radical of vanillyl alcohol (2A, Fig. 6c) reacts with O_2_˙^−^ species to produce the alcohol peroxo species (2B) (P23, Fig. 6c), eventually converting to vanillin and releasing H_2_O_2_ (P24, Fig. 6c). Under these conditions, the catalytic cycle is completed, and the γ-MnO_2_ catalyst is ready for the next catalytic process.

## Conclusions

In summary, we have successfully fabricated manganese oxide-based catalysts with different Mn defects on MnO_2_ with various crystal structures and on γ-MnO_2_ with different amounts of urea. Comprehensive structural characterization highlights the significance of urea in tuning the coordination environment of Mn species in γ-MnO_2_ and the O species in α-MnO_2_. In the gram-scale oxidation of vanillyl alcohol to vanillin, the Mn-defected γ-MnO_2_ catalyst demonstrates excellent photocatalytic performance compared to the O-defected α-MnO_2_ catalyst and other type (β, ε)-MnO_2_ catalysts. DFT calculations and control experiments demonstrate that the defected-Mn species in the γ-MnO_2_ catalyst facilitate the adsorption of vanillyl alcohol and H_2_O, which converts to the corresponding ˙OH species under natural light illustration. The synergistic effect of the Mn-defected species and ˙OH species plays a crucial role in enhancing the photocatalytic performance in the aerobic oxidation of vanillyl alcohol to vanillin over the γ-MnO_2_ catalyst. This study not only presents effective methods for tuning the coordination environments of Mn atoms in MnO_2_ for the selective oxidation of vanillyl alcohol to vanillin but also provides valuable insights for the development of advanced transition metal oxide photocatalysts.

## Author contributions

Q. Ke, Y. Zhang and C. Wan: conceptualization, methodology, and writing – original draft. Y. Zhang, J. Tang, S. Li, X. Guo, T. Hamada, and S. M. Osman: data curation, chemical experiments, and formal analysis. Q. Ke, and C. Wan: funding acquisition and investigation. C. Wan, M. Han, Y. Kang, and Y. Yamauchi: supervision and writing – review & editing.

## Conflicts of interest

There are no conflicts to declare.

## Supplementary Material

SC-015-D3SC05654F-s001
