# Supplementary material for: Sunlight-driven and gram-scale vanillin production via Mn-defected γ-MnO2 catalyst in aqueous environment
Source: Chem Sci. 2024 Feb 19;15(14):5368–75. doi: 10.1039/d3sc05654f (PMC10988585; doi:10.1039/d3sc05654f)
Supplement: SC-015-D3SC05654F-s001 [file SC-015-D3SC05654F-s001.pdf]

This journal is © The Royal Society of Chemistry 2024

1. Supporting experimental details

1.1 Materials

$KMnO_4$ (99.5%, AR), $MnSO_4$•H_2O (99%, AR), $(NH_4)_2S_2O_8$ (98%, AR), vanillin alcohol (98%), urea (99%, AR), and $NaHCO_3$ (99.5%, AR) were purchased from Shanghai Aladdin Biochemical Technology Co., Ltd (China). Ultrapure water (18.2 $M\Omega \cdot cm^{-1}$) was produced by a HOKEE purification system (HOKEE−A1−10, China). Other commercially available reagents, such as alcohols and metal salts, were purchased from TCI (Japan) and used without further purification.

1.2 Synthesis of *γ*−MnO_2

Typically, 1.93 g of $MnSO_4$•H_2O and 1.83 g of $(NH_4)_2S_2O_8$ were dissolved in 30 mL of ultrapure water under vigorous stirring at room temperature for 30 min. Subsequently, *x* mmol of urea (*x*= 0, 1, and 10 mmol) was added and stirred for an additional 30 min to form the MnO_2 precursor solution. The MnO_2 precursor solution was then hydrothermally treated at 90 °C for 24 h. The resulting mixture was filtered, washed three times with ethanol and water alternately, and dried completely at 80 °C for 12 h to obtain 0.8~1 g of a black powder. The black powder is named *γ*-MnO_2(*x*) according to the supplied amount of urea (*x*), and the one without urea is named *γ*−MnO_2. The production cost of the prepared *γ*−MnO_2 catalyst is estimated at £ 29.6/kg, based on the purchase cost of raw materials and energy usage.

1.3 Synthesis of *α*−MnO_2.

Typically, 1.26 g of $KMnO_4$ and 0.72 g of $MnSO_4$•H_2O were dissolved in 80mL of ultrapure water under vigorous stirring at room temperature for 30 min. Subsequently, *x* mmol of urea (*x*= 0, 1, and 10 mmol) was added and stirred for an additional 30 min to form the MnO_2 precursor solution. The MnO_2 precursor solution was then hydrothermally treated at 160 °C for 12 h. The obtained mixture was filtered, washed three times with ethanol and water alternately, and dried completely at 80 °C for 12 h. The product was calcined at 300 °C for 4 h to yield a brown powder. The brown powder is named *α*−MnO_2(*x*) according to the amount of urea added (*x*), and *α*−MnO_2 without urea.

1.4 Synthesis of ***β*−MnO₂**

Typically, 0.632 g of $KMnO_4$ and 2.89g $MnSO_4$•H_2O were dissolved in 80mL of ultrapure water under vigorous stirring at room temperature for 30 min. The MnO_2 precursors were hydrothermally treated at 160 °C for 12 h. The obtained mixture was filtered, washed two or three times with ethanol and water alternately, and then dried at 80 °C for 12 h. The product was calcined at 300 °C for 4h to yield a black powder. The black powder is named β−MnO_2.

1.5 Synthesis of ***ε*−MnO₂**

Typically, 0.169 g of $MnSO_4$•H_2O was dissolved in a mixture of ultrapure water (70 mL) and anhydrous ethanol (7 mL) under vigorous stirring at room temperature. Then 0.84 g $NaHCO_3$ was dissolved in it, stirred for about 10 min, and allowed to stand at room temperature for 3 h. The obtained mixture was filtered, washed two or three times with ethanol and water alternately, and then dried at 80°C for 12 h. The product was calcined at 400°C for 6 h to yield a black powder. The black powder is named ε−MnO_2.

1.6 Catalytic test

In a 10 mL quartz reactor, a mixture of 10 mmol of catalyst, 6 mL of ultrapure water, and 0.77 g of vanillyl alcohol was added. The reactor was operated under an air atmosphere at a temperature of 30 °C, with the reaction temperature controlled using a cycle pump.

The catalytic performance was analyzed using a PANNA A91Plus Gas chromatography (GC) system from China, equipped with a capillary column (HP-5 column, 30 m × 0.32 mm, 0.50 μm film thicknesses). Normalization of the obtained results was carried out using the following equations:

$$Alcohol\ conversion\ (Con.) = \frac{S_{AL} + S_{Others}}{S_{AL} + S_{OL} + S_{Others}} \times 100\% \quad (1)$$

$$Aldehyde\ selectivity\ (Sel.) = \frac{S_{AL}}{S_{AL} + S_{Others}} \times 100\% \quad (2)$$

where S_{AL}, S_{OL}, and S_{Others} refer to the areas of aldehyde, alcohol, and other products in GC, respectively.

1.7 Radical Scavenger Experiments

In a 10 mL quartz reactor, a mixture consisting of 100 mg of catalyst, 2 mL of ultrapure water, 1 mmol of alcohol, and 1 mmol of scavenger (FFA, mannitol, or *p*−BQ) was prepared. The reactor was performed under an air atmosphere at a temperature of 30 °C, with the reaction temperature regulated using a cycle pump.

1.8 Computational Details

The present spin-polarized first principle DFT calculations were performed using Vienna Ab initio Simulation Package (VASP)[1] with the projector augmented wave (PAW) method.[2] The exchange-functional was treated using the generalized gradient approximation (GGA) of Perdew-Burke-Ernzerhof (PBE)[3] functional. The energy cutoff for the plane wave basis expansion was set to 450 eV, and the force on each atom less than 0.02 eV/Å was set as the convergence criterion for geometry relaxation. To avoid interaction between periodic structures, a 15 Å vacuum was added along the Z direction. The Brillouin zone integration was performed using 3×3×1 k-point sampling. The self-consistent calculations employed a convergence energy threshold of 10^{-5} eV. The DFT-D3 method was employed to consider the van der Waals interaction.[4] DFT+U was employed to account for the strong on-site Coulomb repulsion among the localized Mn 3d electrons, using a Mn_{3d} U value of 2.8 eV and a J value of 1.2 eV for Mn ions.[5]

The adsorption energy E_{ads} was calculated according to:

$$E_{ads} = E_{tot} - E_{mol} - E_{sub}$$

where E_{tot} is the total energy of the adsorbed system, and E_{mol} and E_{sub} are the energies of the adsorbed molecule and the substrate, respectively.

1.9 Characterizations

Powder X−ray diffraction (PXRD) spectra were collected on a Smartlab X−ray Polycrystalline Diffraction Analyzer (Rigaku, Japan). Scanning Electron Microscope (SEM) and Transmission Electron Microscope (TEM) images were obtained using JSM−6490LV (JSM, Japan) and Talos F200X microscopes (FEI, USA), respectively. X-

ray photoelectron spectroscopy (XPS) spectra were collected using a PHI-5000C ESCA system (Perkin-Elmer, USA) with Al Kα radiation (hν=1486.6 eV). Ultraviolet-Visible-Near Infrared diffuse reflectance spectroscopy (UV-Vis-NIR DRS) was recorded using a UV-3600plus Spectrometer (Shimadzu, Japan). Electron paramagnetic resonance (EPR) spectra were collected on an EMXPLUS (Bruker, Germany). The EPR analysis was conducted by the addition of 5,5-dimethyl-1-pyrroline N-oxide (DMPO) as a trap for $^{\bullet}OH$ and $O_2^{\bullet-}$ species. For $O_2^{\bullet-}$ species measurement, 5 mg of powder was dispersed in 5 mL of methanol. After ultrasonication for 5 min, 200 μL of the mixed solution was taken and mixed with 200 μL of DMPO solution with a concentration of 100 mM. The mixture was then placed into a capillary tube and tested in the machine at room temperature. Fourier-transform infrared (FT−IR) spectra were recorded on a Nicolet IS50 Fourier Transform Infrared Spectrometer (Thermo Fisher, USA). All *in-situ* diffuse reflectance IR spectra (DRIFTS) were recorded on a Nicolet IS50 FT−IR spectrometer equipped with a DRIFTS cell (OPERANDO−TETRA) and MCT detector. N_2 and O_2 were purged into the cell with a flow rate of ~10 mL•min^{-1} with the catalyst as the background. Water was introduced into the DRIFTS cell *via* the O_2 stream. The catalyst powder was pre-treated in pure N_2 at 30 °C for 30 min before collecting the background.

2. Supplementary results

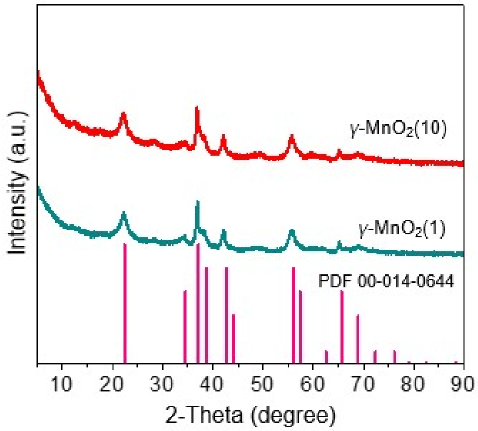

Fig. S1. PXRD patterns of γ−MnO_2(1) and γ−MnO_2(10) particles.

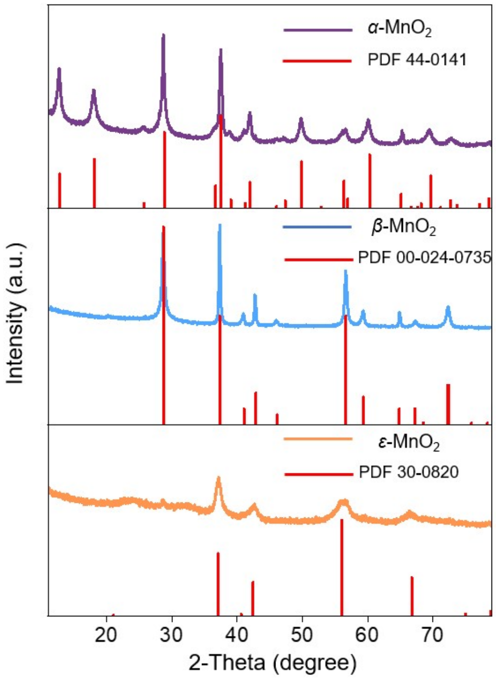

Fig. S2. PXRD patterns of α−MnO_2, β−MnO_2, and ε−MnO_2 particles.

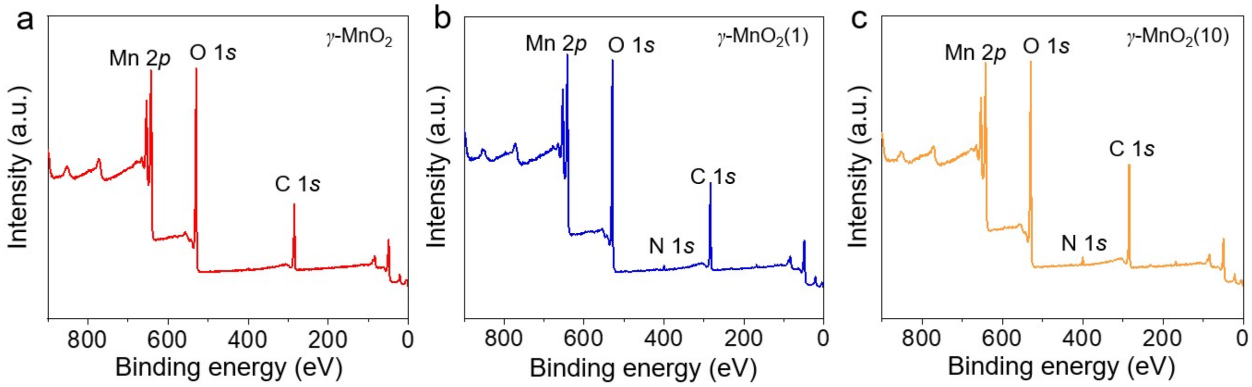

Fig. S3. XPS patterns of (a) γ−MnO_2, (b) γ−MnO_2(1), and (c) γ−MnO_2(10) oxides.

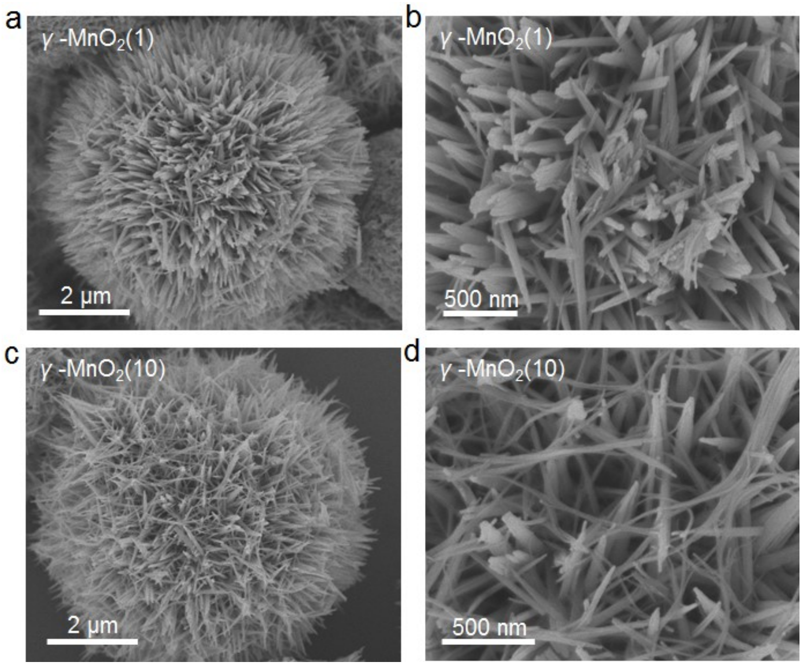

Fig. S4. SEM images of the synthesized (a, b) γ−MnO_2(1) and (c, d) γ−MnO_2(10) particles.

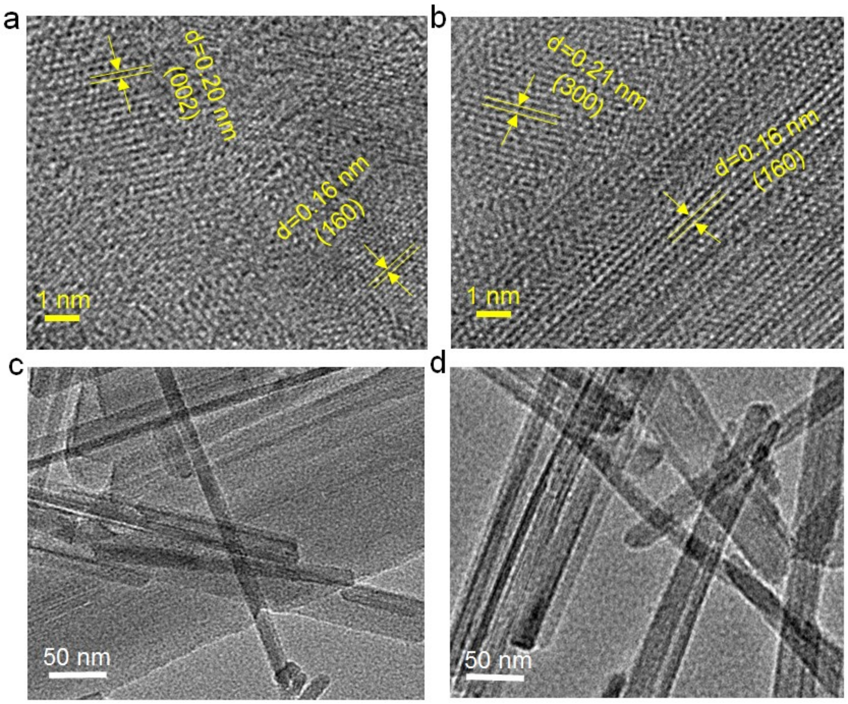

Fig. S5. (a, b) HRTEM and (c, d) TEM images of the synthesized (a, c) $\gamma-MnO_2(1)$ and (b, d) $\gamma-MnO_2(10)$ oxides.

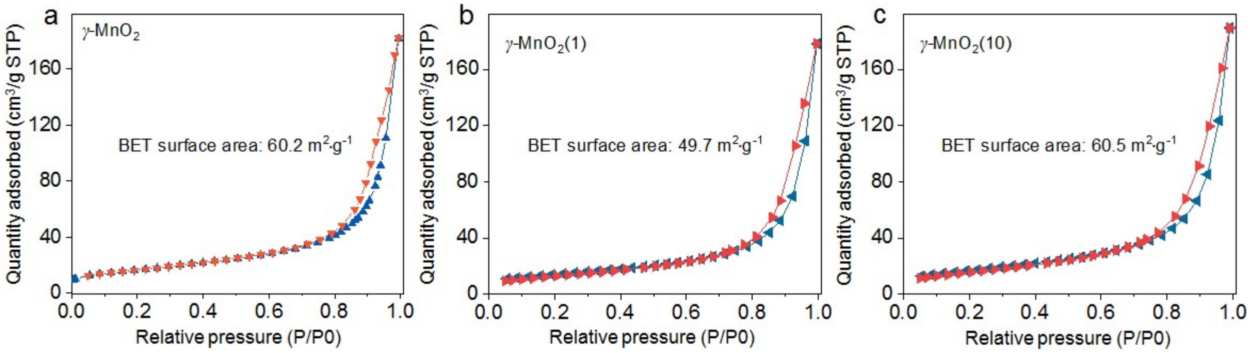

Fig. S6. N_2 adsorption–desorption isotherms of $\gamma-MnO_2$, $\gamma-MnO_2(1)$, and $\gamma-MnO_2(10)$ oxides.

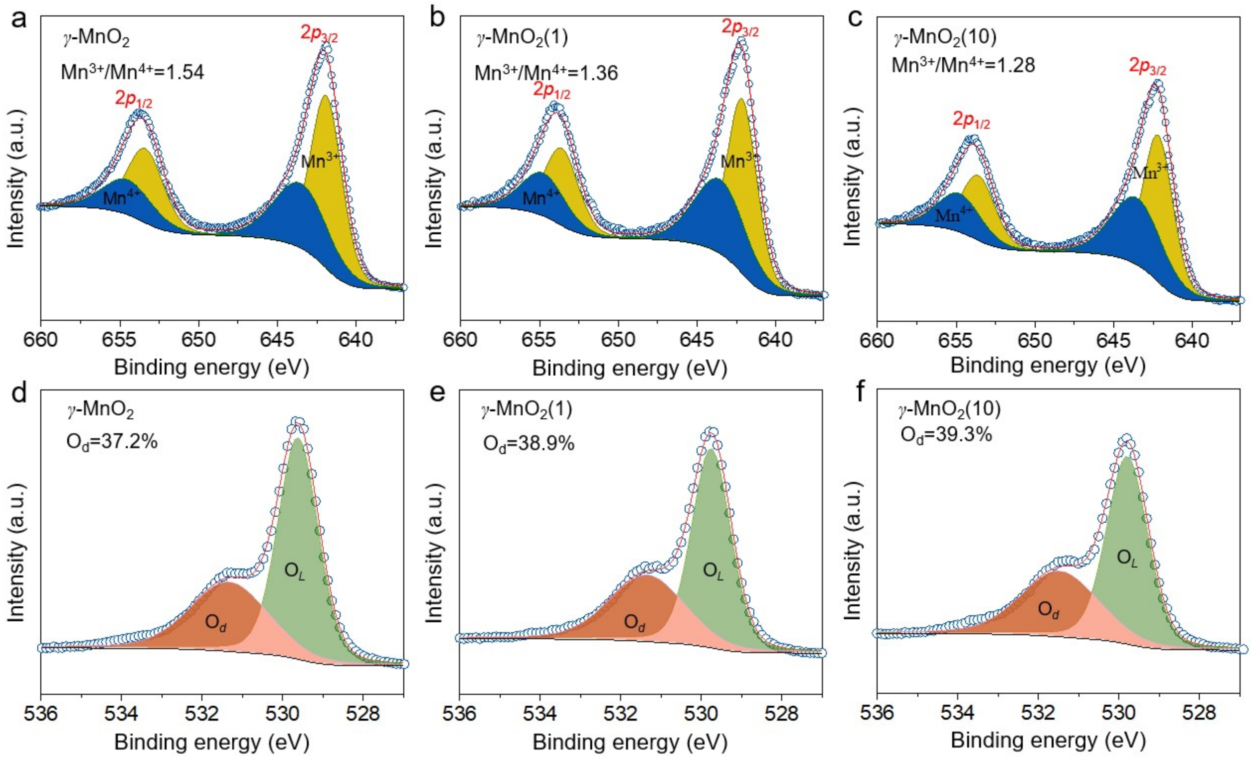

Fig. S7. Mn 2p and O 1s XPS patterns of (a, d) γ−MnO_2, (b, e) γ−MnO_2(1), and (c, f) γ−MnO_2(10) oxides.

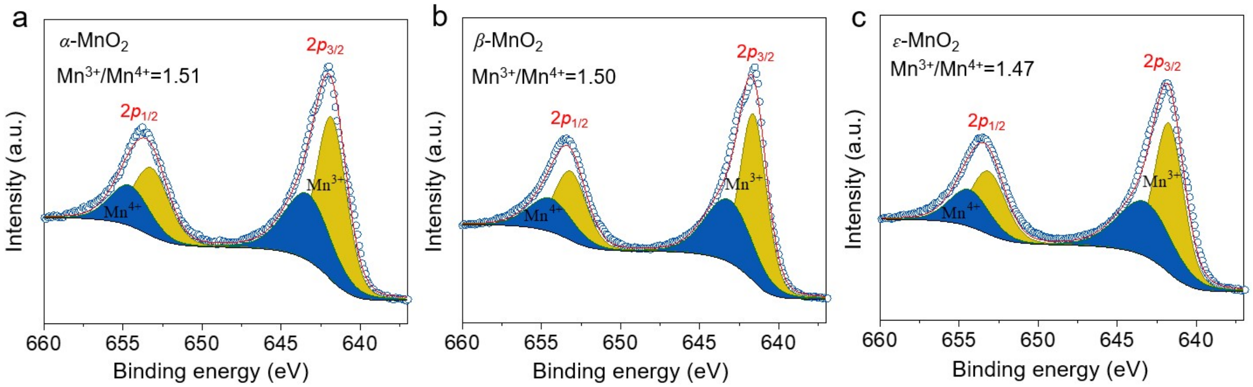

Fig. S8. Mn 2p XPS spectra of (a) α−MnO_2, (b) β−MnO_2, and (c) ε−MnO_2 oxides.

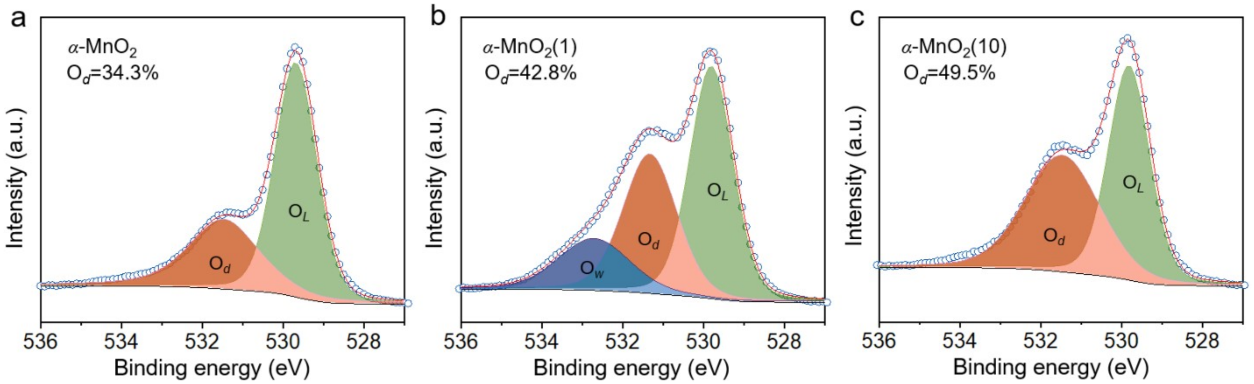

Fig. S9. O 2*p* XPS spectra of (a) *α*−MnO_2, (b) *α*−MnO_2(1), and (c) *α*−MnO_2(10) oxides.

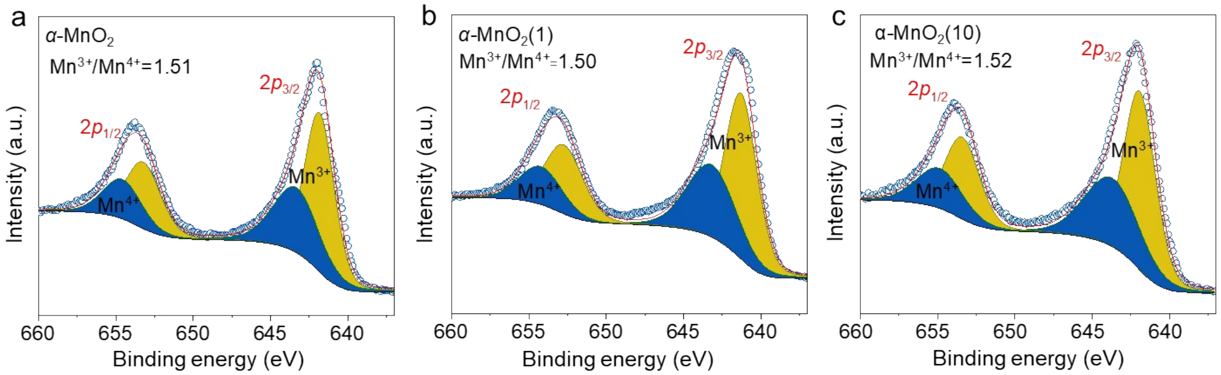

Fig. S10. Mn 2*p* XPS spectra of (a) *α*−MnO_2, (b) *α*−MnO_2(1), and (c) *α*−MnO_2(10) oxides.

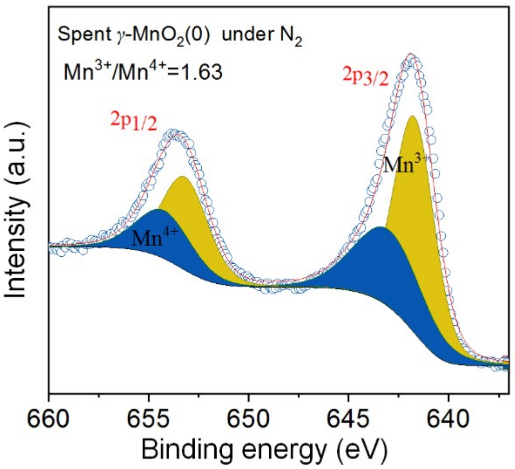

Fig. S11. Mn 2*p* XPS pattern of $\gamma-MnO_2$ used in the oxidation reaction of vanillyl alcohol under N_2 atmosphere.

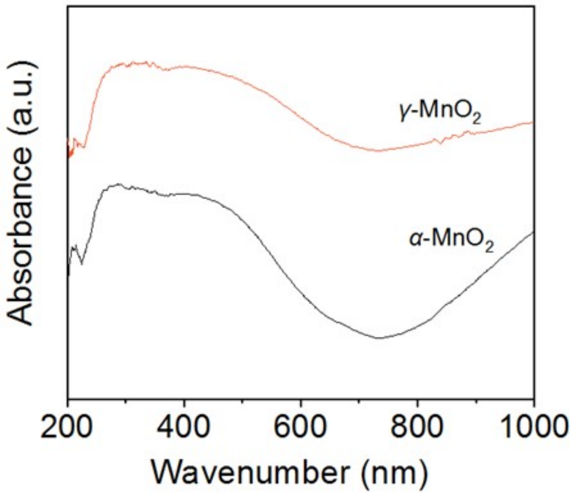

Fig. S12. UV-Vis-NIR DRS of the $\alpha-MnO_2$ and $\gamma-MnO_2$ oxides.

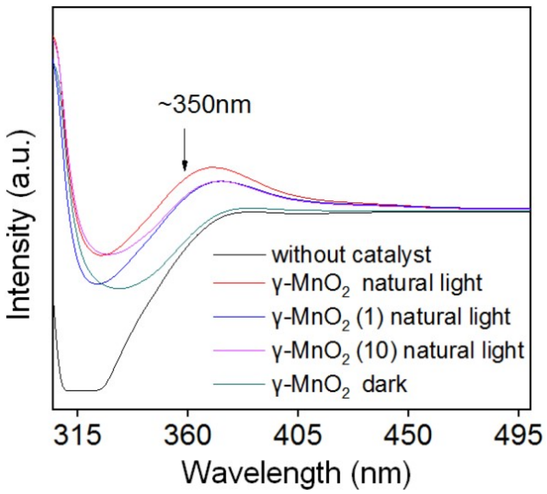

Fig. S13. Detection of H_2O_2 species after oxidation of vanillyl alcohol over various catalysts for 10 min through the iodometry method.

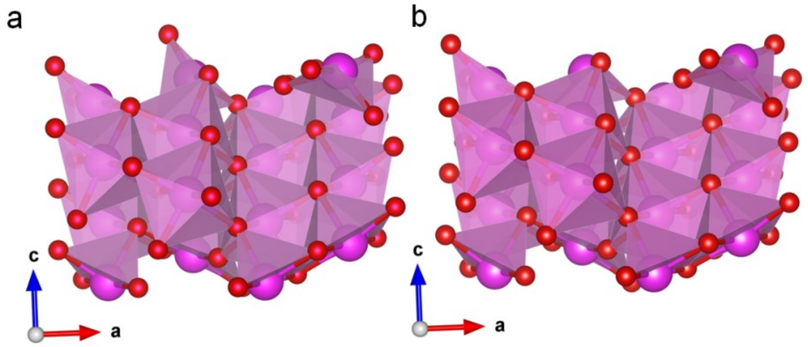

Fig. S14. (a) Perfect (120) facet and (b) defective (120) facet of *γ*−MnO_2 catalyst.

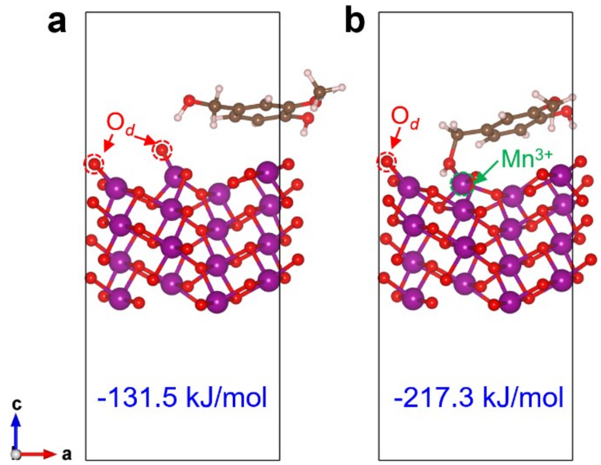

Fig. S15. DFT calculations for the adsorption energy of vanillyl alcohol on γ−MnO_2 without (a) and with (b) surface Mn^{3+} active sites.

Table S1. Comparison of the selected typical studies for the aerobic oxidation of vanillyl alcohol from the CHEM21 Green Metrics Toolkit calculation.

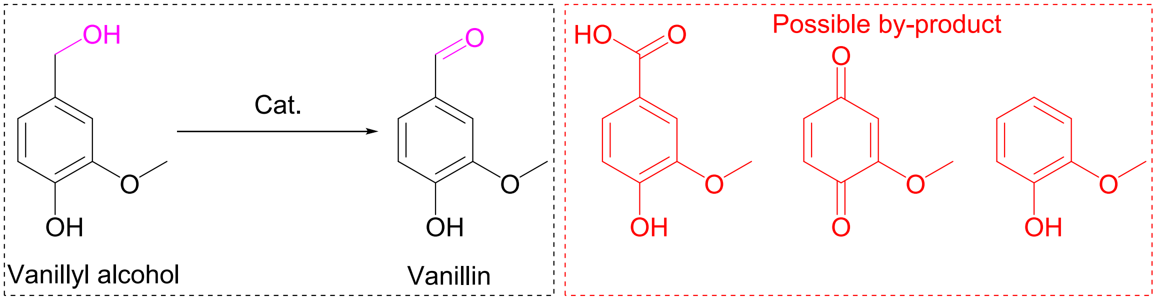

Metric	our study	Ref. 1[6]	Ref. 2[7]	Ref. 3[8]	Ref. 4[9]	Ref. 5[10]	Ref. 6[11]	Ref.7[12]
Solvent	**W**	**O/W**	**O**	**O**	**O**	**W**	**O**	**O/W**
Energy	**R.T.**	**R.T.**	**120**	**R.T.**	**R.T.**	**80**	**120**	**R.T.**
Pressure	**1bar**	**1bar**	**5bar**	**1bar**	**1bar**	**1bar**	**3bar**	**1bar**
work up	√	√	√	—	√	☒	√	√
Con. (%)	**>89**	**>89**	**>89**	**>89**	**<70**	**>89**	**>89**	**>89**
Sel. (%)	**>89**	**>89**	**>89**	**<70**	**<70**	**>89**	**>89**	**>89**
Additive	**Free**	**TEMPO**	**Free**	**Free**	**Free**	**Free**	**Free**	**Free**
Catalyst (toxic,economic)	**Mn**	**Cu**	**Pd**	**Ag**	**Au-Pd**	**Pd**	**AgPd**	**$K_4[Pt_2(P_2O_5H_2)_4]$**

Table S2. Effects of reaction temperatures for the aerobic oxidation of vanillyl alcohol over γ−MnO_2 catalyst.

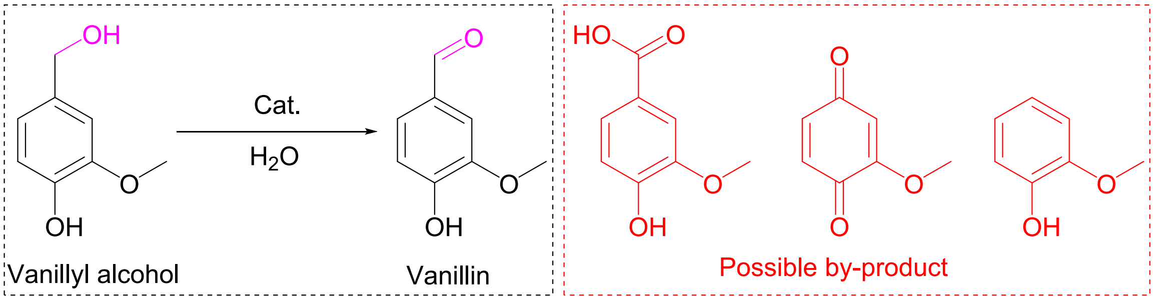

Catalyst	Vanillyl alcohol (mmol)	Temp. (°C)	Con. %	Sel. %
γ−MnO_2	5	20	60.0	87.6
	5	25	69.1	88.7
	5	30	93.4	95.7
	5	35	82.0	88.8

Table S3. Effects of reaction solvents for the aerobic oxidation of vanillyl alcohol over γ−MnO_2 catalyst (T = 30 °C).

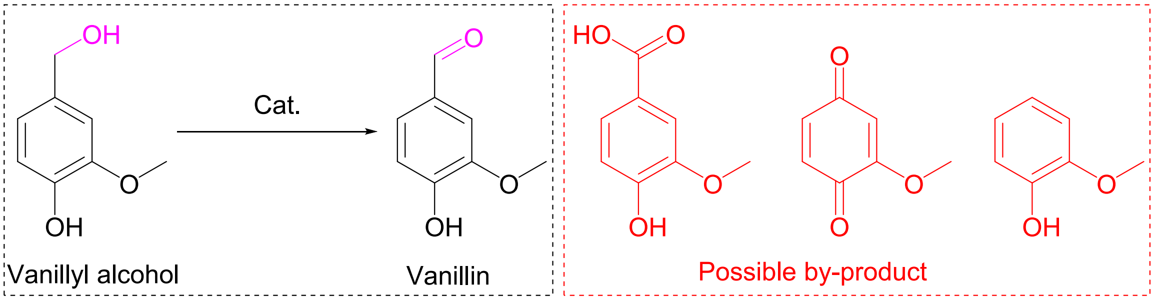

Catalyst	Vanillyl alcohol (mmol)	Solvent	Con. %	Sel. %
γ−MnO_2	5	MeCN	43.4	63.8
	5	DMF	29.5	46.8
	5	Toluene	79.5	21.7
	5	MeOH	16.3	44.0
	5	1,4-Dioxane	18.7	46.9
	5	H_2O	93.4	95.7

Reference

1. G. Kresse and J. Furthmüller, *Comp. Mater. Sci.*, 1996, **6**, 15-50.
2. P. E. Blöchl, *Phys. Rev. B*, 1994, **50**, 17953.
3. M. Ernzerhof and J. P. Perdew, *J. Chem. Phys.*, 1998, **109**, 3313-3320.
4. S. Grimme, J. Antony, S. Ehrlich and H. Krieg, *J. Chem. Phys.*, 2010, **132**, 154104.
5. X. Li, J. Ma, C. Zhang, R. Zhang and H. He, *J. Environ. Sci.*, 2020, **91**, 43-53.
6. N. C. Jana, S. Sethi, R. Saha and B. Bagh, *Green Chem.*, 2022, **24**, 2542-2556.
7. W. Sun, S. Wu, Y. Lu, Y. Wang, Q. Cao and W. Fang, *ACS Catal.*, 2020, **10**, 7699-7709.
8. J. Estrada-Pomares, S. Ramos-Terrón, G. Lasarte-Aragonés, R. Lucena, S. Cárdenas, D. Rodríguez-Padrón, R. Luque and G. de Miguel, *J. Mater. Chem. A*, 2022, **10**, 11298-11305.
9. M. Wu, J.-H. Pang, P.-P. Song, J.-J. Peng, F. Xu, Q. Li and X.-M. Zhang, *New J. Chem.*, 2019, **43**, 1964-1971.
10. W. Fu, L. Yue, X. Duan, J. Li and G. Lu, *Green Chem.*, 2016, **18**, 6136-6142.
11. W. Sun, P. Lin, Q. Tang, F. Jing, Q. Cao and W. Fang, *Catal.Sci. Technol.*, 2021, **11**, 7268-7277.
12. J.-J. Zhong, W.-P. To, Y. Liu, W. Lu and C.-M. Che, *Chem. Sci.*, 2019, **10**, 4883-4889.
